# Belatacept Maintenance Immunosuppression for Calcineurin Inhibitor Sparing or Avoidance in Pancreas Transplant Recipients With Progressive Renal or Renal Allograft Dysfunction

**DOI:** 10.1111/ctr.70310

**Published:** 2025-09-24

**Authors:** Jeanne M. Chen, Richard S. Mangus, Asif A. Sharfuddin, John A. Powelson, Muhammad S. Yaqub, Muhammad Y. Jan, Andrew J. Lutz, Jonathan A. Fridell

**Affiliations:** ^1^ IU Health Department of Pharmacy Indianapolis USA; ^2^ IU Health/Indiana University School of Medicine Department of Surgery Indianapolis USA; ^3^ IU Health/Indiana University School of Medicine Department of Nephrology Indianapolis USA

**Keywords:** belatacept, immunosuppression, pancreas transplant

## Abstract

Belatacept may be used to spare or replace calcineurin inhibitors (CNI) to preserve renal function. Use in pancreas transplant (PTx) is limited by increased risk of pancreas rejection. This retrospective analysis included all PTxs performed between 2004 and 2023. A 1:2 case/control analysis was performed to identify predictors of belatacept use and compare allograft and patient survival. Of 731 PTxs, 21 (3%) started belatacept (eight simultaneous pancreas and kidney (SPK), three pancreas after kidney (PAK), and 10 pancreas transplant alone (PTA). At 1 year, Δ estimated glomerular filtration rate (eGFR) was +7% SPK, −15% PAK, and +32% PTA. Case–control analysis found no demographic predictors for belatacept except older recipient age for PTA. No difference in median kidney, pancreas, or patient survival was observed compared to control. Pancreas rejection occurred in two SPKs. There were two death censored pancreas allograft failures, both PTAs. Kidney allografts failed in two SPK and one PAK. Eight patients died. Six were still receiving belatacept at time of death with functioning allografts. Belatacept use after PTx is safe and can provide some renal recovery. Belatacept was initiated with eGFR approaching 20 mL/min/1.73m^2^. Earlier introduction may result in better outcomes.

**Abbreviations**
CMV: cytomegalovirus
CNI: calcineurin inhibitor
ESRD: end stage renal disease
GFR: glomerular filtration rate
MPA: mycophenolic acid
mTORi: mammalian target of rapamycin
PAK: pancreas after kidney transplant
PTA: pancreas transplant alone
Ratg: rabbit antithymocyte globulin
Sir: sirolimus
SPK: simultaneous pancreas and kidney
Tac: tacrolimus

## 1 Introduction

Pancreas transplantation offers a unique opportunity to achieve insulin independence and stable normoglycemia in carefully selected patients with insulin dependent diabetes mellitus. Given that the procedure involves a major abdominal surgery and necessitates lifelong immunosuppression, it is most frequently performed in conjunction with another solid organ transplant, typically a kidney transplant for individuals with diabetic nephropathy progressing to end stage renal disease. In such cases, the pancreas and kidney can be transplanted simultaneously from the same deceased donor, referred to as simultaneous pancreas kidney transplantation (SPK), or sequentially, as in pancreas after kidney transplantation (PAK), when a living kidney donor is available. In addition to these combined procedures, isolated pancreas transplantation remains an option for individuals experiencing severe diabetes related complications, such as hypoglycemia unawareness, despite optimal medical therapy [1, 2].

Modern immunosuppression regimens for pancreas transplant recipients typically include induction with a t‐cell depleting agent followed by maintenance immunosuppression including a calcineurin inhibitor (CNI), most often tacrolimus (Tac) in combination with an adjunct medication such as mycophenolic acid (MPA) (including either mycophenolic acid or mycophenolate mofetil) or mammalian target of rapamycin inhibitors (mTORi) such as sirolimus (Sir) or everolimus, with or without long term steroids [3, 4]. These regimens are associated with consistently low rejection rates and excellent long‐term patient and allograft survival rates [5, 6, 7, 8, 9]. Despite excellent outcomes, the potential side effects of these maintenance medications are considerable and can be difficult to manage. In particular, CNIs can be associated with progressive nephrotoxicity, especially when used in combination with an mTORi. Unfortunately, there are currently no ideal oral immunosuppression alternatives to CNIs that achieve similar freedom from rejection and long‐term allograft survival. Alternative or supplemental long‐term maintenance immunosuppression for rescue in these cases is necessary and to date have not been well described. In recent years, some initial success using the costimulatory blockage agent belatacept as a CNI sparing or avoidance strategy for pancreas transplant recipients has been described including three small case series of 2–8 pancreas transplant recipients and a single multicenter randomized control trial [10, 11, 12, 13, 14]. This strategy is somewhat limited because it is not an option for all recipients, particularly as this medication is contraindicated in Epstein Barr virus seronegative patients.

This study is a single center retrospective analysis of all the recipients where belatacept was used as maintenance immunosuppression in order to decrease or discontinue CNIs following pancreas transplant in scenarios where the recipient was developing progressive native renal (for PTA) or renal allograft (for SPK or PAK) dysfunction, and no alternative options were apparent. Please appreciate that in all cases, without adequate baseline immunosuppression, these recipients were perceived to be at significantly increased risk for allograft rejection or loss if not provided with some form of alternative adequate maintenance immunosuppression strategy, and all available alternatives had been exhausted. Belatacept was initiated in all cases with the intention that this would serve as a long‐term component of maintenance immunosuppression and, in fact, many of the subjects were successfully maintained using this strategy for many years.

## 2 Methods

The medical records for all adult deceased donor pancreas transplants performed at Indiana University between January 1, 2004 and December 31, 2023, were reviewed (*n* = 712). This study was approved by the Indiana University Institutional Review Board. Data were extracted from the comprehensive transplant recipient registry maintained at our center, individual electronic and written medical records, and the original donor medical history. Inclusion criteria included all PAK, PTA, or SPK recipients that received at least 3 months of belatacept therapy with at least 1 year of follow‐up. As there was not a control cohort of patients that deviated from standard immunosuppression and not initiated on belatacept rescue maintenance therapy, study patients receiving belatacept instead of standard therapy were compared to a group of recipients that tolerated standard immunosuppression using a 1:2 case–control study methodology which was designed to identify demographic predictors for belatacept indications. The case and controls were matched manually for transplant type (SPK, PAK, or PTA), gender, age (±10 years), race, year of transplant (±5 years), donor age (±10 years), and donor cause of death. Statistical analysis of the groups was performed using Chi‐square and analysis of variance with *p* value significant at the 0.05 level. The SPK, PAK, and PTA groups were analyzed separately. Kaplan–Meier methodology was employed to compare study groups for long‐term survival, including pancreas allograft and patient survival. For the SPK group, kidney allograft survival was analyzed in the same manner. For the PAK group, renal allograft survival was calculated from the time of pancreas transplant regardless of the timing of the previous kidney transplant. Patients in the PTA group were considered to have renal failure with need for a kidney transplant, dialysis, or with patient death. For each transplant type, a contemporaneous control cohort is provided for comparison. These three control groups included all patients transplanted during the same time period, had the same type of transplant, but did not receive belatacept. Patients with a re‐transplant of the pancreas were excluded from the control groups in all cases.

All recipients were listed for transplantation according to standard protocols and procedures as established by our center and the Organ Procurement and Transplantation Network (OPTN). During the study period, the potential recipient had to be insulin dependent, and the majority had a fasting serum C‐peptide level <2 ng/mL. For PTA, the recipients had to demonstrate preserved renal function, usually with a creatinine clearance of at least 50 mL/min/1.73 m^2^.

Pancreas allografts were procured following aortic flush with preservation solution and topical cooling with saline slush and prepared on the back bench with standard donor iliac artery Y‐graft reconstruction for arterial inflow as previously described [15, 16, 17]. The recipient operation was performed through a midline incision with the pancreas allograft positioned with the head and duodenum oriented upward and the tail toward the pelvis. Systemic venous drainage was performed to the vena cava or to the right common iliac vein. Arterial perfusion of the allograft was typically established from the right common iliac artery. All pancreas allografts were drained enterically using a stapled technique as described elsewhere [18]. For SPK, the pancreas and kidney were placed ipsilaterally on the right side as described previously [19].

The induction immunosuppression protocol consisted of five daily doses of rabbit antithymocyte globulin (rATG) (1 mg/kg/dose) initiated prior to allograft implantation and a maintenance regimen of Tac and Sir initiated on postoperative Day 1. Goal trough levels for Tac were 6–8 mCg/mL and Sir 3–6 mCg/mL [20, 21]. If Sir was not tolerated, full dosage MPA in the form of either mycophenolate mofetil (1000 mg po bid) or Mycophenolic acid (720 mg po bid) was used as a substitute. For PTA recipients, MPA in the form of either mycophenolate mofetil (500 mg po bid) or Mycophenolic acid (360 mg po bid) was added to the standard immunosuppression protocol [22]. On some occasions where MPA was not tolerated, azathioprine was used as an alternative. Steroids were used exclusively as a premedication for rATG and were discontinued following induction in all recipients. In situations where one of the standard maintenance immunosuppression medications was poorly tolerated or if augmentation of baseline immunosuppression seemed necessary, a monthly intravenous infusion of basiliximab (40 mg) was added [23]. Patients receiving belatacept infusion received a dose of 5 mg/kg every 2 weeks for five doses then 5 mg/kg every 4 weeks. All recipients received routine preoperative antibiotics, prophylaxis against cytomegalovirus (CMV) with oral valganciclovir and prophylaxis against *Pneumocystis jirovecii* pneumonia with trimethoprim and sulfamethoxazole, unless contraindicated. Systemic anticoagulation was not routinely used unless the patient had a specific history of a coagulation disorder. All recipients were started on aspirin immediately postoperatively. Prophylaxis for deep venous thrombosis with subcutaneous heparin or enoxaparin was initiated on post‐operative Day 1. Sequential compression devices were maintained until prophylaxis was initiated.

Pancreas transplant biopsy was infrequently pursued. A diagnosis of pancreas rejection was made based on clinical parameters with elevated serum amylase and/or lipase in which other possible causes were ruled out. Recipients that had episodes of pancreas rejection were identified as those that had received treatment with either corticosteroid bolus and/or a t‐cell depleting agent outside of induction immunosuppression. SPK recipients in whom kidney rejection was suspected underwent biopsy of the kidney allograft for diagnosis of rejection.

## 3 Results

**FIGURE 1:**
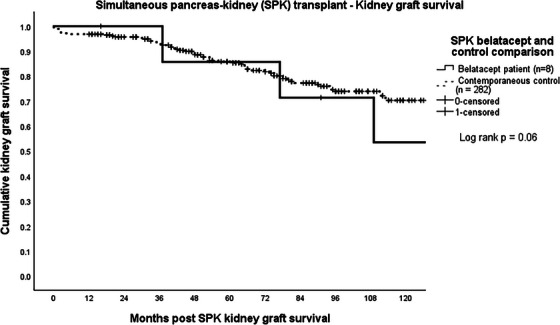
Simultaneous pancreas‐kidney (SPK) transplant – Kidney graft survival.

**FIGURE 2:**
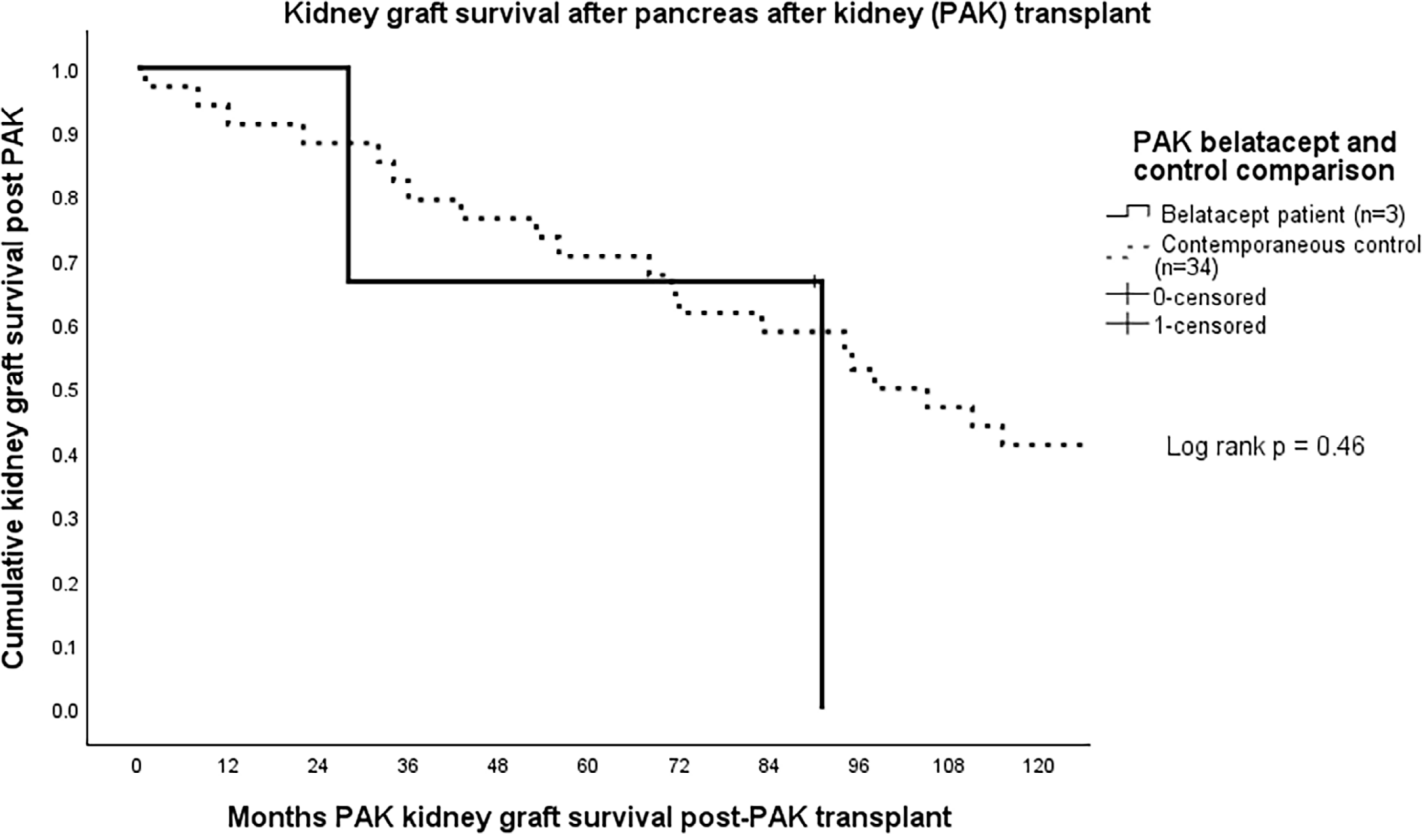
Kidney graft survival after pancreas after kidney (PAK) transplant.

**FIGURE 3:**
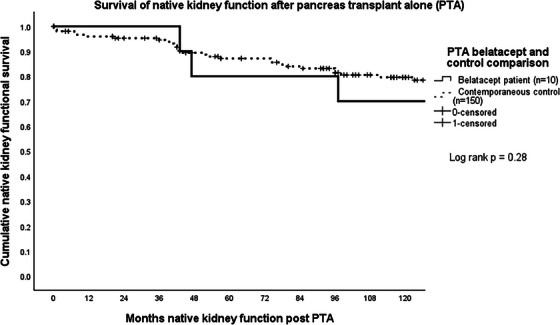
Survival of native kidney function after pancreas transplant alone (PTA).

Of 712 pancreas transplants performed between January 1, 2004 and December 31, 2023 (325 SPK, 106 PAK, 281 PTA), 21 recipients (3%) were initiated on belatacept including eight SPK, three PAK, and 10 PTA with first patient receiving belatacept in 2012. All were started on belatacept to spare or eliminate CNI in the setting of progressive renal insufficiency. An additional four patients that received belatacept were excluded from the analysis. One pancreas retransplant after PAK received belatacept for less than 3 months when it was discontinued due to severe fatigue. One SPK developed pancreas allograft failure and was subsequently initiated on belatacept for the remaining kidney allograft.  Two SPK recipients were excluded because they also received complement C5 blocking agents (eculizumab and ravulizumab) for atypical HUS. Donor and recipient demographics and outcomes for the 21 recipients studied, the contemporaneous recipients of the same type of pancreas transplant, and the 1:2 matched controls are shown for SPK, PAK, and PTA recipients separately in Table 1. Belatacept completely replaced CNI in one SPK, and six PTA recipients and was used to spare CNI in seven SPK, three PAK, and four PTA. Median eGFR was 23 mL/min/1.73 m^2^ at the time of transition with a median eGFR of 16.5 mL/min/1.73 m^2^ for SPK, 27 mL/min/1.73 m^2^ for PAK, and 23 mL/min/1.73 m^2^ for PTA (Table 1). Median months to conversion to belatacept was 85 (67 for SPK, 161 for PAK, and 74 for PTA). Initiation of belatacept in SPK occurred <1 year in two patients, 1–5 years in two patients, at 5–10 years in three patients and >10 years in one patient. Three PTA recipients started belatacept between 2‐ and 5‐years following transplant; three at 5–10 years and treatment started more than 10 years following transplant in three recipients. All three PAK initiated belatacept at 13 years following transplant. Estimated median pancreas allograft and patient survival were not different between the contemporaneous control and belatacept groups. Estimated kidney graft survival in SPK and PAK as well as native kidney survival in PTA were also not different between groups (Table 1, Figures 1, 2, 3). One‐year after starting belatacept, median ΔeGFR was +7% for SPK, −15% for PAK, and +32% for PTA. Case–control analysis found no demographic predictors for indications for belatacept in SPK or PAK. Older recipient age (51 years vs. 42 years) was predictive for need of belatacept for PTA recipients. Pancreas rejection occurred in two SPK recipients at 3 months after belatacept initiation, both of which responded to methylprednisone bolus and taper. There were two death censored pancreas allograft failures, both of which were PTA recipients; one patient required allograft pancreatectomy for donor derived sarcoma [24], and a second developed pancreas allograft failure 9 months after stopping belatacept when HD was initiated. This was the only PTA that went on to develop ESRD. Kidney rejection occurred in one PAK recipient who underwent kidney retransplant 3 years after the pancreas transplant. He was switched from CNI 11 years after the second kidney transplant, developed steroid resistant Banff 1a rejection 1 year later which responded to treatment with corticosteroids and rATG. He did to return to dialysis 1 year after rejection treatment, at which point belatacept was stopped and CNI was resumed. The pancreas continues to function at 15 years following transplant. Two additional kidney allografts failed in two SPKs at 6 and 12 months after belatacept started. One PTA was initiated on dialysis (as mentioned above), and another received a kidney after PTA transplant. Two opportunistic infections occurred; CMV in a PTA recipient which responded to treatment with valganciclovir and *P. jirovecii* in an SPK. In addition to the patient that developed sarcoma, one PTA recipient developed squamous cell carcinoma. Eight patients (four PTA, three SPK, one kidney after PTA) died of intestinal hemorrhage, myocardial infarction, trauma, three pneumonia (due to COVID, *P. jirovecii*, and *Pseudomonas aeruginosa)* and two of unknown causes. Six of these were on belatacept at time of death with functioning allografts.

**TABLE 1:** Case‐control comparison (1:2) of simultaneous pancreas and kidney transplant (SPK), pancreas after kidney transplant (PAK), and pancreas transplant alone recipients receiving belatacept conversion (*n* = 8, 3, and 10, respectively) to those remaining on standard immunosuppression (*n* = 16, 6, and 20, respectively).

	Case‐control analysis											
Simultaneous pancreas and kidney (SPK)	All SPK contemporaneous [number (%)]^#^	Belatacept conversion	Standard immunosuppression	*p*‐value	All PAK contemporaneous [number (%)]^#^	Belatacept conversion	Standard immunosuppression	*p*‐value	All PTA contemporaneous [number (%)]^#^	Belatacept conversion	Standard immunosuppression	*p*‐value
Number	282	8	16		34	3	6		150	10	20	
***Demographics***												
Gender												
Male	169 (60%)	3	6		25 (74%)	1	2		35 (46%)	3	6	1.00
Female	115 (40%)	5	10		9 (26%)	2	4		42 (54%)	7	14	
Race												
White	240 (85%)	6	12		32 (94%)	3	6		71 (92%)	8	16	1.00
Other	44 (15%)	2	4		2 (6%)	0	0		6 (8%)	2	4	
Age at transplant												
Mean/median (SE)	44 / 43 (0.6)	39 / 40 (3.2)	38 / 36 (1.6)	0.85	47 / 46 (1.3)	51 / 53 (5.5)	50 / 50 (1.6)	0.77	40 / 40 (1.3)	50 / 51 (3.4)	44 / 42 (1.9)	0.08
Recipient body mass index												
Mean/median (SE)	25.9 / 25.5 (0.3)	26.9 / 27.0 (1.3)	26.5 / 26.3 (1.0)	0.79	27.4 / 26.5 (0.8)	21.6 / 21.1 (0.8)	23.4 / 22.8 (1.5)	0.46	27.0 / 24.9 (0.6)	27.6 / 27.2 (1.0)	28.7 / 28.4 (2.4)	0.61
Donor age												
Mean/median (SE)	26 / 25 (0.6)	23 / 18 (5.9)	26 / 21 (3.7)	0.62	27 / 24 (2.0)	42 / 45 (4.8)	41 / 41 (2.7)	0.86	26 / 24 (1.2)	29 /25 (4.9)	26 / 26 (2.4)	0.57
GFR at transplant												
Mean/median (SE)	21 / 10 (5.0)	10 / 10 (0)	27 / 11 (7.1)	0.11	54 / 60 (4.4)	47 / 46 (7.5)	57 / 60 (5.3)	0.30	65 / 60 (2.4)	61 / 60 (3.8)	67 / 60 (3.1)	0.25
Time to belatacept conversion (months)												
Mean/median (SE)	n/a	62 / 67 (18)	n/a		n/a	162 / 161 (1.9)	n/a		n/a	79 / 74 (15)	n/a	
***Outcomes***												
**Rejection of pancreas**												
Within first year	17 (6%)	2 (25%)	1 (6%)	0.25	1 (3%)	0 (0%)	0 (0%)	n/a	2 (3%)	0 (0%)	1 (5%)	1.00
Any		4 (50%)	4 (25%)	0.36		0 (0%)	0 (0%)			1 (10%)	5 (25%)	0.33
Months to first rejection (*n* = 14)												
Mean/median (SE)		41 / 38 (21)	30 / 23 (16)	0.70		n/a	n/a	n/a		113 / 113 (0)	53 / 53 (17)	0.22
**Any rejection of kidney**												
**Estimated median survival (months (SE))** ^*^												
Pancreas allograft	145 (4)	144 (18)	168 (19)	0.55	130 (17)			0.04	163 (34)	163 (25)	157 (19)	0.80
Kidney allograft (or native kidney)	147 (4)	116 (20)	158 (21)	0.23	118 (13)	70 (24)	83 (15)	0.36	198 (8)	170 (26)	187 (18)	0.32
Patient	160 (4)	144 (18)	179 (18)	0.36	157 (32)			0.04	172 (8)	170 (23)	182 (17)	0.32

* Using Kaplan‐Meier analysis

^#^ For years 2009 to 2023

Immunosuppression regimens used with and without belatacept are shown in Table S1. All recipients except one received triple drug therapy including belatacept with the exception of one PTA who was intolerant of other agents prior to belatacept (MPA associated gastrointestinal side effects and neutropenia with azathioprine). Belatacept was used in combination with low dose CNI in 14 patients. Two recipients received cyclosporine in place of tacrolimus for neurotoxicity and thrombotic microangiopathy, respectively. CNI withdrawal occurred in seven patients. Mycophenolic acid was the most common adjunct agent used but was replaced with sirolimus or azathioprine in patients who developed gastrointestinal side effects. One recipient received maintenance corticosteroid therapy.

Belatacept was well tolerated following introduction; no patient discontinued belatacept due to side effects or adverse events. Overall, 10 patients remain on belatacept at the end of the study period, none of which require HD (five PTA, two PAK, three SPK). Current immunosuppression regimens are described in Table S2. All but four are maintained on a low dose CNI in combination with belatacept (tacrolimus *n* = 5, cyclosporine *n* = 1). The remaining four recipients in whom CNI has been withdrawn are receiving sirolimus with belatacept along with low dose mycophenolate in three of the four. Three PTAs have been receiving belatacept for >10 years and the remaining two have been on treatment for 4 and 6 years. The two PAK recipients have received belatacept for 4 years and all three SPKs continue belatacept at 1 year after initiation.

## 4 Discussion

Progressive renal dysfunction following pancreas transplantation remains an important concern. In SPK and PAK recipients, maintenance of long‐term renal allograft function is critical for overall patient and pancreas allograft survival, while in PTA recipients, the progression of renal impairment can lead to late pancreas allograft loss and the need for dialysis or kidney transplantation. The management of immunosuppression in this population is especially challenging due to the relatively high incidence of chronic allograft rejection associated with pancreas transplantation. As such, pancreas transplantation is often managed with a more consistently heavy‐handed immunosuppression regimen than other abdominal organ transplants in order to maintain long‐term allograft survival, which in turn increases the potential for adverse effects, particularly chronic renal injury. Balancing effective rejection prophylaxis while minimizing long term toxicity remains a challenge. The widespread use of CNI as the cornerstone of maintenance immunosuppression has contributed to decreased overall allograft loss to rejection but long‐term use is associated with nephrotoxicity manifesting as arteriolar hyalinosis, interstitial fibrosis, and progressive glomerulosclerosis [25]. As pancreas transplant recipients require lifelong immunosuppression, strategies to mitigate CNI‐associated nephrotoxicity while maintaining adequate immunosuppression are essential.

Belatacept, a selective T‐cell costimulation blocker targeting the CD80‐CD86 pathway has emerged as a potential CNI sparing alternative, particularly in kidney transplant, where it has demonstrated improved long‐term renal function and a favorable cardiovascular and metabolic profile [26, 27, 28]. However, experience with belatacept in pancreas transplantation remains limited as recent studies have shown that, although belatacept can reduce CNI nephrotoxicity, it carries an increased risk of acute rejection in certain transplant populations, particularly in regimens that do not include low dose CNIs. The phase II multicenter randomized trial sponsored by the National Institute of Health conducted in SPK recipients was halted early as belatacept initiated at the time of transplant without CNI provided insufficient immunosuppression to reliably prevent pancreas rejection in SPK transplants undergoing tacrolimus withdrawal [13]. However, belatacept used with low dose CNI or mTORi in SPK or PTA recipients with renal dysfunction or severe gastroparesis has resulted in improvement in kidney function with no acute rejection episodes reported [11, 12]. A more recent report by Perrier et al. of a case series of four SPK and one PTA patients with longer term follow‐up demonstrated that belatacept can successfully be used in preserving good pancreas and renal function in pancreas transplant recipients recommending that the optimal use of belatacept conversion protocols include continuing low dose CNI [14]. These findings suggest that belatacept based regimens can be safely used in pancreas transplant recipients, potentially improving renal function without compromising pancreatic allograft survival.

This single center, retrospective case–control study is the largest reported experience to date evaluating the use of belatacept in pancreas transplant recipients, particularly in the context of chronic kidney disease and the need for CNI minimization or withdrawal. Out of 712 pancreas transplants performed over a 20‐year period, 21 recipients (3%) were transitioned to a belatacept based regimen after belatacept was approved for use in kidney transplantation by the US Food and Drug Administration in 2011. Our results suggest that belatacept is a feasible and generally well tolerated alternative to CNI based immunosuppression in select pancreas transplant patients, with variable impacts on graft function depending on transplant type.

Belatacept was primarily introduced in the setting of progressive renal dysfunction, with a median estimated GFR at initiation of 23%. The most notable renal recovery was observed among PTA recipients who demonstrated a median improvement of +32% in GFR at 1 year. SPK recipients experienced modest improvement (+7%) and PAK showed a median decline, although numbers were small, and these recipients were the furthest out from transplant. Furthermore, renal allograft survival was calculated from the time of pancreas transplantation, so the kidney allograft had already been implanted for some period of time prior to the initiation of the survival timing. Overall, the finding of improvement in GFR and similar preservation of renal function was remarkable considering that all of the recipients were initiated on treatment with belatacept with pending allograft failure as a genuine concern. It is important to note that in this cohort of patients, belatacept initiation occurred late after transplant with an eGFR approaching 20 mL/h. Despite these encouraging results, it would certainly be reasonable to consider starting belatacept even earlier, particularly in PTA recipients. Notably, older recipient age was associated with the need for belatacept in PTA recipients, a factor that may reflect longer exposure to diabetes prior to transplant with higher likelihood of irreversible diabetes related native kidney nephropathy.

Importantly, pancreas graft function was preserved in the majority of cases following belatacept initiation. Only two death‐censored pancreas graft losses occurred, both were PTA recipients. One case was unrelated to immunosuppression (donor derived sarcoma) and the other occurred after discontinuation of belatacept. Episodes of pancreas rejection were infrequent and responded to standard therapy, supporting the potential efficacy of belatacept in preventing rejection in pancreas recipients. These observations align with growing experience in kidney transplantation, where belatacept has been shown to reduce chronic nephrotoxicity while maintaining low rates of acute rejection in selected patients [26, 27, 28].

Kidney allograft outcomes in this cohort were more variable, with three allograft losses observed—two SPK recipients and one PAK. The latter developed steroid resistant rejection following late conversion, highlighting the potential risks associated with belatacept in high‐risk patients. This underscores the need for careful selection and monitoring, particularly in recipients with a history of multiple transplants or prior rejection. Importantly, 10 patients remained on belatacept at the end of follow‐up, none of whom required dialysis, suggesting that with appropriate patient selection, long term renal preservation is achievable.

Belatacept was well tolerated in this cohort. No patients discontinued belatacept due to adverse events. Opportunistic infections were rare and treatable with one case each of Cytomegalovirus and *P. jirovecii* pneumonia. Two malignancies occurred – one donor derived sarcoma and one squamous cell carcinoma. Although causality cannot be definitively linked to belatacept, monitoring for malignancy remains important. Of note, eight deaths occurred during the study period, largely due to non‐immunologic causes such as cardiovascular disease and trauma. Notably, all six patients who were on belatacept at the time of death had functioning allografts, suggesting that death was not related to allograft failure.

One of the key strengths to the study is the duration of follow‐up which extends beyond 10 years in several recipients. This long‐term perspective offers a unique contribution to the limited literature on belatacept use in pancreas transplantation, where most prior literature focused on short term outcomes or isolated case reports. In addition, to our knowledge this is the first series to include a case–control group comparison, allowing for more meaningful comparison of outcomes among transplant recipients. This approach strengthens the internal validity of our findings and allows for a more clinically meaningful comparison of outcomes of belatacept treated patients and those maintained on CNI based regimens offering clearer insight into the potential benefits and risks of belatacept based immunosuppression in pancreas transplantation.

Nevertheless, our study does have limitations. The retrospective, single center design and cohort presented was relatively small and heterogeneous with respect to type of transplant and timing of the change. In an attempt to include all patients who received belatacept, a mixture of SPK, PTA, and PAK were included. Second, heterogeneity in immunosuppression regimens and timing of belatacept initiation may confound interpretation of outcomes. As a single center study, the findings may be influenced by institutional practices and clinician discretion, which could have affected both patient selection and management strategies.

Despite these limitations, this study adds important preliminary evidence supporting the use of belatacept in pancreas transplant recipients with declining renal function or CNI intolerance. Most patients continued to maintain allograft function and avoided dialysis, even after long term follow‐up. Belatacept may be especially beneficial in PTA recipients, in whom long term nephrotoxicity from CNI poses a significant risk to renal function.

In conclusion, our findings along with recent case reports suggest that belatacept used with or without low dose CNI can be a safe and effective strategy for preserving pancreas graft function while optimizing renal function in pancreas transplant recipients. Advantages to belatacept over other immunosuppressants include a once every 4 weeks administration maintenance regimen as well as a favorable side effect profile as it does not cause nephrotoxicity, gastrointestinal side effects, or bone marrow suppression. Conversely, one primary challenge to utilizing belatacept therapy is the high cost of therapy. Prior authorization by the insurance company is frequently required for this “off label” indication in pancreas transplantation. Even with approval, the cost of copayments may be unaffordable for some patients. Confirming adequate insurance coverage and ensuring affordable copays is imperative prior to initiating therapy to maximize adherence. These findings support further investigation of costimulation blockade‐based regimens as part of individualized, renal sparing immunosuppression strategies in pancreas transplantation.

Immunosuppression following pancreas transplantation presents several challenges, including the need for lifelong therapy, increased risk of infections, and adverse drug effects that can impair long term graft function. Notably, CNIs are effective but nephrotoxic, raising concern for renal injury over time. Efforts to minimize immunosuppression burden, such as steroid withdrawal for example, can heighten rejection risk and may require more potent maintenance regimens. Personalizing therapy to reduce toxicity is critical to improving outcomes, and in select cases, we have successfully used belatacept as a renal sparing alternative and basiliximab as a replacement for other adjunct agents in patients who experienced intolerable side effects [23, 29]. Both of these strategies support a tailored approach to immunosuppression.

## Conflicts of Interest

The authors of this manuscript have no conflicts of interest to disclose as described by Clinical Transplantation. We acknowledge participation in the Transplant Peer Review Network and complied with the journal's author guidelines and policies.

## Supporting information




**Supplementary Table 1**. Immunosuppressant Regimens (n=21)

**Supplementary Table 2**. Recipients Continuing Belatacept Therapy (n=10)

## Data Availability

The data that support the findings of this study are available from the corresponding author upon reasonable request.
