# Supplementary material for: Belatacept Maintenance Immunosuppression for Calcineurin Inhibitor Sparing or Avoidance in Pancreas Transplant Recipients With Progressive Renal or Renal Allograft Dysfunction
Source: Clin Transplant. 2025 Sep 24;39(10):e70310. doi: 10.1111/ctr.70310 (PMC12457987; doi:10.1111/ctr.70310)
Supplement: Supplementary file 1 — Supplementary Table 1. Immunosuppressant Regimens (n=21) [file CTR-39-e70310-s001.docx]

Supplementary Table 2. Immunosuppressant Regimens (n=21)

| Transplant Type | Regimen Pre Belatacept | Belatacept Regimen |
| --- | --- | --- |
| 5 PTA | Tac/Sir/MPA | Bela/Sir/MPA |
| 1 PTA, 3 SPK | Tac/Sir/MPA | Bela/Tac/MPA |
| 1 PTA | Tac/Sir/MPA | Bela/Sir |
| 1 PTA | Tac/Sir/Aza | Bela/Tac/Pred |
| 1 SPK | Tac/Sir/Bas | Bela/Tac/Sir |
| 1 PTA, 1 PAK | Tac/Sir | Bela/Tac/MPA |
| 1 SPK | Tac/Sir | Bela/Sir/MPA |
| 1 SPK | Tac/Sir | Bela/Tac/Sir |
| 2 PAK | Tac/MPA | Bela/Tac/Aza |
| 1 SPK | Tac/MPA | Bela/Tac/MPA |
| 1 PTA | CSA/MPA | Bela/CSA/Aza |
| 1 SPK | CSA/Sir/Aza | Bela/CSA/Sir |
