# Supplementary material for: Belatacept Maintenance Immunosuppression for Calcineurin Inhibitor Sparing or Avoidance in Pancreas Transplant Recipients With Progressive Renal or Renal Allograft Dysfunction
Source: Clin Transplant. 2025 Sep 24;39(10):e70310. doi: 10.1111/ctr.70310 (PMC12457987; doi:10.1111/ctr.70310)
Supplement: Supplementary file 2 — Supplementary Table 2. Recipients Continuing Belatacept Therapy (n=10) [file CTR-39-e70310-s002.docx]

Supplementary Table 3. Recipients Continuing Belatacept Therapy (n=10)

|  | Transplant Type | Time Post Transplant (years) | Duration of Belatacept Therapy (years) | Current eGFR  (mL/min/1.73m^2^) | Current Immunosuppression Regimen |
| --- | --- | --- | --- | --- | --- |
| 1 | PTA | 16 | 10 | 59 | Belatacept/Sir/MPA |
| 2 | PTA | 10 | 10 | 37 | Belatacept/Sir/MPA |
| 3 | PTA | 13 | 10 | 56 | Belatacept/Sir/MPA |
| 4 | PTA | 17 | 6 | 38 | Belatacept/Sir |
| 5 | PTA | 8 | 4 | 20 | Belatacept/Tac//Prednisone |
| 6 | SPK | 11 | 1 | 20 | Belatacept/Tac/MPA |
| 7 | SPK | 3 | 1 | 25 | Belatacept/Tac/Sir |
| 8 | SPK | 1 | 1 | 78 | Belatacept/CSA/Sir |
| 9 | PAK | 17 | 4 | 30 | Belatacept/Tac/Aza |
| 10 | PAK | 17 | 4 | 43 | Belatacept/Tac/MPA |
